# Quantitative omics analyses of NCOA4 deficiency reveal an integral role of ferritinophagy in iron homeostasis of hippocampal neuronal HT22 cells

**DOI:** 10.3389/fnut.2023.1054852

**Published:** 2023-01-19

**Authors:** Emily F. Bengson, Cole A. Guggisberg, Thomas W. Bastian, Michael K. Georgieff, Moon-Suhn Ryu

**Affiliations:** ^1^Department of Food Science and Nutrition, College of Food, Agricultural and Natural Resource Sciences, University of Minnesota, Saint Paul, MN, United States; ^2^Department of Pediatrics, Medical School, University of Minnesota, Minneapolis, MN, United States; ^3^Department of Food and Nutrition, College of Human Ecology, Yonsei University, Seoul, Republic of Korea

**Keywords:** ferritinophagy, proteomics, RNA-seq, ferritin, iron deficiency, IRP2

## Abstract

**Introduction:**

Neurons require iron to support their metabolism, growth, and differentiation, but are also susceptible to iron-induced oxidative stress and cytotoxicity. Ferritin, a cytosolic iron storage unit, mediates cellular adaptation to fluctuations in iron delivery. NCOA4 has been characterized as a selective autophagic cargo receptor facilitating the mobilization of intracellular iron from ferritin. This process named ferritinophagy results in the degradation of ferritin and the consequent release of iron into the cytosol.

**Methods:**

Here we demonstrate that NCOA4 is important for the adaptation of the HT22 mouse hippocampal neuronal cell line to cellular iron restriction. Additionally, we determined the pathophysiological implications of impaired ferritinophagy *via* functional analysis of the omics profile of HT22 cells deficient in NCOA4.

**Results:**

NCOA4 silencing impaired ferritin turnover and was cytotoxic when cells were restricted of iron. Quantitative proteomics identified IRP2 accumulation among the most prominent protein responses produced by NCOA4 depletion in HT22 cells, which is indicative of functional iron deficiency. Additionally, proteins of apoptotic signaling pathway were enriched by those responsive to NCOA4 deficiency. Transcriptome profiles of NCOA4 depletion revealed neuronal cell death, differentiation of neurons, and development of neurons as potential diseases and bio functions affected by impaired ferritinophagy, particularly, when iron was restricted.

**Discussion:**

These findings identify an integral role of NCOA4-mediated ferritinophagy in the maintenance of iron homeostasis by HT22 cells, and its potential implications in controlling genetic pathways of neurodevelopment and neurodegenerative diseases.

## 1. Introduction

Iron is an essential nutrient required for a variety of molecular and metabolic processes, including cell development, DNA replication, respiration, and energy production. In humans, iron deficiency remains among the most prevalent nutrient disorders worldwide and causes anemia, impaired motor and cognitive function, increased risk of significant psychopathologies, including depression, anxiety, schizophrenia, and autism spectrum disorder, and other health defects (1–3). On the other hand, excess iron within cells can facilitate the formation of harmful reactive oxygen species (ROS), and thus cause cytotoxicity (4). Hence, the supply, storage, and distribution of iron must be tightly regulated to maintain cellular and systemic iron homeostasis within an ideal range that is physiologically adequate and not imposing harm.

Ferritin is a cytosolic protein for iron storage, composed of 24 ferritin-H (FTH) and ferritin-L (FTL) subunits, which form a highly stable spherical configuration accommodating up to 4,500 atoms of iron. FTH and FTL protein translation responds to the cytosolic labile iron pool (LIP), which is constantly gauged by iron responsive element (IRE)-binding proteins, IRP1 and IRP2 (4). IRPs sterically hinder the translation of ferritin transcripts, particularly, when cellular iron levels become limited. When the LIP expands, IRPs dissociate and promote the translation of ferritin subunits. IREs are also present in the transcripts of iron importers transferrin receptor 1 (*TFRC*) and divalent metal transporter 1 (*SLC11A2*), and the iron exporter ferroportin 1 (*SLC40A1*), which allows a coordinated regulation of iron import, export, and storage for maintenance of cellular iron homeostasis.

Recently, an IRP-independent mechanism regulating ferritin iron release *via* selective autophagy has been identified. This process, called ferritinophagy, is initiated by the binding of an autophagic cargo receptor protein, nuclear receptor coactivator 4 (NCOA4), to iron-laden ferritin (5, 6). The transfer of NCOA4-ferritin to the lysosome by ferritinophagy results in the proteolysis of ferritin, and, in turn, the release of its iron content. NCOA4 is upregulated in response to cellular iron restriction (7, 8). The transcript of NCOA4 does not contain an IRE and is not regulated by IRP activity (6), and NCOA4 is post-translationally controlled by iron status (7, 8).

The physiological implications of NCOA4-mediated ferritinophagy have been determined using various *in vitro* and *in vivo* models of NCOA4 deficiency, particularly those recapitulating cell-types and tissues integral to systemic iron metabolism. Of relevance are enterocytes (9), hepatocytes (10), macrophages (5, 11, 12), and erythroid progenitors (7, 13, 14), which function in tissues carrying out iron absorption, storage, recycling, and utilization, respectively. NCOA4 in these cell-types are essential for maintaining cellular iron availability and for supporting nutritional and physiological demand for iron elevated by either dietary iron restriction or red blood cell production. Systemic deletion of *Ncoa4* in mice leads to functional iron deficiency, manifested with hematological signatures of iron deficiency anemia despite iron overload in tissues (15).

Dysregulation of ferritin and intracellular iron in neurons have been implicated in neurological disorders (16). Moreover, a potential role of NCOA4 in neuropathy has been recently proposed (17). Thus, we investigated the regulation and roles of NCOA4 in a hippocampal neuronal cell line model, HT22 cells. HT22 is an immortalized mouse hippocampus-derived neuronal cell line extensively studied as a model of not only cholinergic neurons, but also glutamate excitotoxicity and endogenous oxidative stress. The latter has been implicated in various neurodegenerative diseases, thus making the HT22 cell line of physiological relevance (18, 19). Our findings confirmed the requirement of NCOA4-mediated ferritin turnover for the survival of HT22 cells under iron restriction. Moreover, functional analyses of the omics profiles of NCOA4 deficiency in HT22 cells revealed enrichment of gene responses associated with diseases and biological functions of brain cells. These support a possible role of ferritinophagy in the pathophysiology of neurodegenerative diseases, previously hypothesized (17).

## 2. Materials and methods

### 2.1. Cell culture and chemical treatments

HT22 cells (a generous gift from Dr. Phu Tran from the University of Minnesota, MN, USA) were cultured in Dulbecco’s Modified Eagle’s Medium (DMEM) with 4.5 g/L glucose and L-glutamine without sodium pyruvate, supplemented with 10% fetal bovine serum and 1% penicillin-streptomycin (Corning, Manassas, VA). Cells were maintained at a confluency of less than 70% in 5% CO_2_ at 37°C. To produce cellular iron overload and deprivation, cell cultures were treated with ferric ammonium citrate (FAC; Sigma-Aldrich, St. Louis, MO) and deferoxamine (DFO; Sigma-Aldrich, St. Louis, MO), respectively. Final concentrations of each chemical are provided in the section “3. Results” and corresponding figure legends.

### 2.2. siRNA-mediated gene silencing

NCOA4 levels were manipulated by siRNA-mediated gene silencing. Cells were plated to maintain confluency at less than 70% and transfected using Lipofectamine RNAiMAX following the manufacturer’s instructions (Thermo Fisher Scientific, Waltham, MA). Briefly, siRNA stock solutions at 20 μM were diluted with Opti-MEM to 150 nM, and then added to an equal volume of a solution prepared by mixing Lipofectamine RNAiMAX and Opti-MEM at a 1:50 ratio. After incubation at room temperature for 5 min, the siRNA-Lipofectamine complexes were administered to cells to yield a final siRNA concentration of 12.5 nM. Cells were then incubated in 5% CO_2_ at 37°C for up to 48 h. *Ncoa4* siRNA (Silencer Select ID s77517, Ambion, Waltham, MA) and negative control siRNA (4390847) (Ambion, Waltham, MA) were used in all knockdown experiments as described and validated previously (8, 13).

### 2.3. RNA isolation and quantitative PCR (qPCR)

Cells were harvested, washed with ice-cold phosphate-buffered saline (PBS), and centrifuged at 200 × *g* for 10 min at 4°C. RNA was isolated using the TRI reagent (Sigma-Aldrich, St. Louis, MO) following the manufacture’s protocol. Briefly, cells were lysed with TRI reagent at room temperature for 5 min and treated with 0.1 volume of 1-bromo-chloropropane. The top clear RNA phase was collected after separation by centrifugation at 12,000 × *g* for 15 min at 4°C. RNA was precipitated with 2-propanol by centrifugation at 12,000 × *g* for 10 min at 4°C, washed with 75% ethanol, and centrifuged at 7,500 × *g* for 5 min at 4°C. RNA yield and purity were determined spectrophotometrically using a Beckman Coulter DU 730 Life Science UV/Vis spectrophotometer. Equal amounts of total RNA (150 ng) were reverse transcribed using a High-Capacity cDNA Reverse Transcription Kit (Applied Biosystems, Waltham, MA), and 1/10 dilutions of cDNA samples were PCR amplified using PowerUP SYBR Green Master Mix reagent (Applied Biosystems, Waltham, MA) combined with gene-specific primers designed using the NCBI Primer-BLAST tool (20). Relative gene expression levels were determined by the ΔΔCt method and normalized to *Tbp* levels. Primers for each gene are listed in Supplementary Table 1.

### 2.4. Protein extraction and western blot analyses

Cell pellets were lysed using an NP-40-based lysis buffer [100 mM Tris-HCl (pH 7.5), 50 mM KCl, 0.1% NP40, 5.0% glycerol, water, and protease inhibitor cocktail (Roche, Basel, Switzerland)], centrifuged at 19,000 × *g* for 5 min at 4°C, and the supernatants were collected for cellular protein analyses. Protein contents of cell lysates were determined by bicinchoninic acid assay (Thermo Fisher Scientific, Waltham, MA). For western blot analyses, equal amounts of protein (15–30 μg) were denatured by boiling in Laemmli buffer supplemented with 2.5% 2-mercaptoethanol for 10 min. Proteins were separated by SDS-PAGE using 4–15% TGX gels (Bio-Rad, Hercules, CA), and transferred to nitrocellulose membranes using a Trans-Blot Turbo Transfer system (Bio-Rad, Hercules, CA). Protein transfer was confirmed *via* Ponceau Red staining. For detection of specific proteins, membranes were blocked with 5% milk in PBS for 45 min at room temperature and incubated with primary antibodies anti-NCOA4 (A302-272A, Bethyl Laboratories, Montgomery, TX) at 1:1,000, anti-ferritin (F502, Sigma-Aldrich, St. Louis, MO) at 1:2,000, and anti-GAPDH (12004167, Bio-Rad, Hercules, CA) at 1:10,000. The anti-IRP2 IgG was a generous gift from Dr. Betty Leibold at the University of Utah and was used at 1:1,000. After washes with PBS containing 0.1% Tween-20, proteins were visualized using relevant near-infrared fluorescence-conjugated secondary antibodies applied at 1:10,000 and an Odyssey Fc imager (Li-Cor, Lincoln, NE). Protein abundance from western images was quantified using the Li-Cor Image Studio Lite software and normalized to corresponding GAPDH values.

### 2.5. Cell viability and bioenergetics assays

Iron is critical for neuronal viability and to support their high energy production. Cell density and neuronal viability were determined *via* trypan blue exclusion and a dehydrogenase-activity-mediated colorimetric assay Cell Counting Kit-8 (CCK-8; Sigma-Aldrich, St. Louis, MO) following the manufacturer’s instructions. In brief, equal number of cells were seeded and treated on a 96-well plate. CCK-8 solution (10 μL) was added to each well and cells were incubated for 2 h in 5% CO_2_ at 37°C. Absorbance at 450 nm was determined using a BioTek Synergy H1 microplate reader. For cellular bioenergetics measures, HT22 cells were transfected with siRNA as described above. Iron deficiency compromises cellular respiration. Forty-eight hours after siRNA treatment, real-time oxygen consumption rates (OCR) and extracellular acidification rates (ECAR) were simultaneously measured using a Seahorse XFe24 Extracellular Flux Analyzer (Agilent Technologies; Santa Clara, CA). Measurements were taken at baseline and after treatments with 1 μM oligomycin (ATP synthase inhibitor), 3 μM FCCP (eliminates mitochondrial proton gradient allowing maximal ETC electron flow and oxygen consumption), and 1 μM antimycin A combined with 1 μM rotenone (inhibitors of electron transport chain complex III and I, respectively) as described (21, 22). Mitochondrial-specific basal respiration, ATP-coupled respiration, maximal respiration, spare respiratory capacity, coupling efficiency were calculated as described (22). Prior to bioenergetics assays, cultures were treated with 1μM Hoechst 33342 and nuclei were imaged with fluorescent microscopy using a ZEISS Celldiscoverer 7 as we have described (23). The OCR and ECAR data were then normalized to cell density for each well.

### 2.6. Quantitative proteomics analysis

Snap-frozen cells were processed at the University of Minnesota Center for Mass Spectrometry and Proteomics (CMSP) for proteomic analyses using the Tandem Mass Tag (TMT) system (Thermo Fisher Scientific, Waltham, MA). At the CMSP, cell pellets were reconstituted with extraction buffer [7 M urea, 2 M thiourea, 0.4 M triethylammonium bicarbonate at pH 8.5, 20% acetonitrile and 4 mM tris(2-carboxyethyl)phosphine], sonicated, and processed using a Barocycler NEP2320 (Pressure Biosciences, Inc., South Easton, MA). After treatment with 8 mM iodoacetamide, protein contents were determined by Bradford assay. Subsequently, samples were digested with trypsin (Promega, Madison, WI) at a 1:40 ratio of total protein for 16 h at 37°C. Digested samples were freeze-dried, cleaned using an Extract Clean C18 SPE cartridge (Grace-Davidson, Deerfield, IL), and resuspended in 0.1 M triethylammonium bicarbonate, pH 8.5, to yield 1 μg protein/μL. Equal amount of each sample (20 μg) were labeled with TMT Isobaric Label Reagent (Thermo Scientific, Watham, MA) per manufacturer’s protocol. Labeled samples were resuspended in 20 mM ammonium formate, pH 10, in 98:2 water:acetonitrile and fractionated offline by high pH C18 reversed-phase chromatography as previously described (24). Peptide-containing fractions were dried *in vacuo*, resuspended in 2% acetonitrile and 0.01% formic acid, and analyzed using an Orbitrap Fusion mass spectrometer (Thermo Scientific, Watham, MA). Final analysis of proteomics data was carried out using Scaffold 5 (Proteome Software, Portland, OR).

### 2.7. RNA-sequencing (RNA-seq)

Total RNA for RNA-seq was isolated from TRI-reagent-treated cells using the Direct-zol RNA Miniprep Kit (Zymo Research, Irvine, CA) following the manufacturer’s instructions. Sample quality assessment, library creation, and next-generation sequencing were carried out at the University of Minnesota Genomics Center. Total RNA was quantified using a fluorometric RiboGreen assay, and RNA integrity was assessed using an Agilent BioAnalyzer 2100. The RIN of all samples were within the range of 7.9 to 8.9. Total RNA samples were converted to sequencing libraries using Takara Bio’s SMARTer Stranded Total RNA-Seq – Pico Mammalian Kit following the manufacturer’s protocol. Then, indexed libraries were normalized and pooled for sequencing. Libraries were sequenced on an Illumina NextSeq 550 instrument to achieve more than 10 million 75-bp paired-end reads per sample. The edgeR software package (25) was used for normalization and differential expression analysis of the RNA-seq data. The sequencing dataset reported in this paper has been deposited in the Gene Expression Omnibus (GEO) database (accession no. GSE211931).

### 2.8. Bioinformatic and statistical analyses

Functional enrichment analyses of the quantitative proteomics data were conducted using the Database for Annotation, Visualization, and Integrated Discovery (DAVID) functional annotation tools (26) and Gene Set Enrichment Analysis (GSEA) (27). Pathway analyses of differential expressions revealed by the transcriptome analyses were conducted using the Ingenuity Pathway Analysis (IPA) software package (Qiagen). All data are presented as mean ± standard deviation. Based on the experimental design and number of comparisons, statistical significance was determined using either Student’s *t*-tests, or with *post-hoc* tests of one-way or two-way ANOVA. Dunnett’s post-test and Bonferroni post-test were performed for one-way and two-way ANOVA, respectively. Statistical analyses were performed using the JMP Pro 14 software (SAS Institute, Cary, NC), and *P*-values lower than 0.05 were considered to indicate statistically significant differences.

## 3. Results

### 3.1. NCOA4 responds to iron status in the HT22 mouse hippocampal neuronal cell line

**FIGURE 1:**
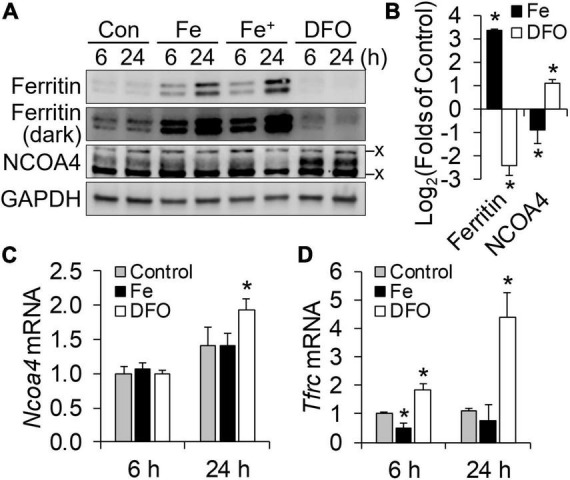
NCOA4 post-transcriptionally responds to iron availability in HT22 mouse hippocampal neuronal cells. HT22 cells were treated with ferric ammonium citrate (Fe, 200 μM; Fe^+^, 400 μM) and desferrioxamine (DFO, 100 μM) for cellular iron overload and restriction, respectively. **(A)** Responses of ferritin and NCOA4 to iron availability, measured by western blot analyses. x, non-specific bands. **(B)** Quantitation of ferritin and NCOA4 western blots. HT22 cells were treated with Fe or DFO for 24 h (*n* = 4 independent experiments). Protein abundance was normalized to GAPDH. **(C,D)** Effects of cellular iron status on transcript abundance of *Ncoa4* **(C)** and *Tfrc* **(D)**, normalized to *Tbp* levels in HT22 cells (*n* = 3 biological replicates). Data are presented as mean ± SD (*n* = 3 biological replicates). *P < 0.05, compared with control levels.

The responsiveness of NCOA4 to iron status can vary by cell-type and developmental stage (8). Thus, we first assessed whether iron status regulates NCOA4 in the immortalized HT22 mouse hippocampal cell line. Cellular iron restriction by the iron chelator DFO increased NCOA4 protein levels in HT22 cells as early as 6 h after treatment (Figures 1A, B). Supplemental iron at either 200 or 400 μM reduced NCOA4 abundance after 24 h, but not after 6 h. Notably, ferritin levels increased within 6 h of iron supplementation, i.e., without a change in NCOA4 levels. After 24 h of iron treatment, NCOA4 responded to changes in iron levels in a direction opposite to that of ferritin. Because iron added at 200 and 400 μM were producing comparable effects on NCOA4 and ferritin expression, we selected the lower dose of iron treatment for our subsequent studies. *Ncoa4* mRNA abundance remained stable (Figure 1C) despite changes in *Tfrc* mRNA levels by cellular iron restriction and overload (Figure 1D). These results support a role of NCOA4 in facilitated ferritin turnover, and its regulation by neuronal iron status at a post-transcriptional level (7, 8).

### 3.2. NCOA4 facilitates ferritin turnover and survival of HT22 cells during iron deficiency

**FIGURE 2:**
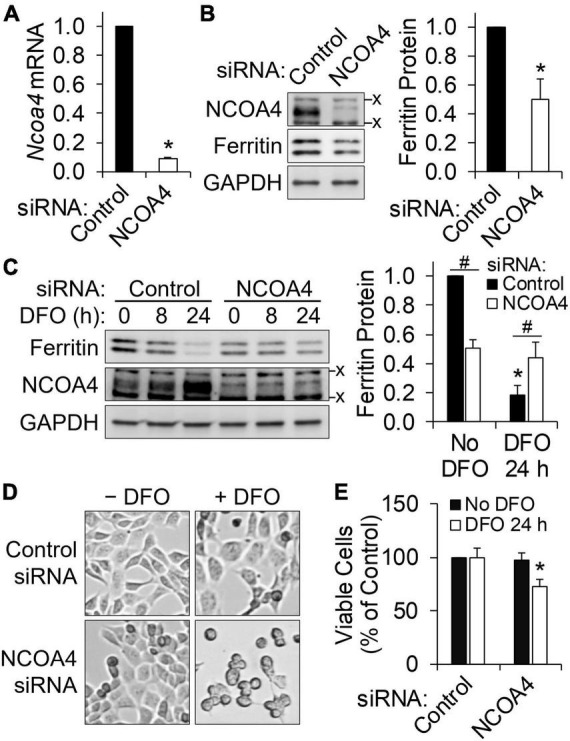
HT22 neuronal cells require NCOA4 for ferritin turnover and survival during iron restriction. NCOA4 was knocked-down *via* liposome-mediated siRNA delivery, and DFO (100 μM) was added to induce iron deficiency. **(A)** siRNA-induced *Ncoa4* knockdown confirmed by qPCR, normalized to *Tbp* levels. **(B)** Loss in ferritin protein by NCOA4 depletion. Ferritin quantitation was normalized to GAPDH. **(C)** NCOA4 deficiency impairs the repression of ferritin by iron restriction. *P < 0.05 by DFO; ^#^*P* < 0.05 by NCOA4 depletion. **(D,E)** Morphological changes **(D)** and reduced viability **(E)** of NCOA4-depleted HT22 neuronal cells by iron restriction. Data presented as mean ± SD of *n* = 3–6 independent experiments. *P < 0.05 compared with control levels. x, non-specific band. siRNA, small interfering RNA; qPCR, quantitative real-time PCR.

Deferoxamine is a cell-impermeable iron chelator and thus produces iron deficiency by limiting iron for cellular import. We hypothesized that NCOA4-mediated ferritinophagy would function as an alternative iron source when neuronal iron uptake is limited. To test this, HT22 cells were transfected with siRNA to silence NCOA4. Successful depletion of NCOA4 by RNAi was confirmed at mRNA (Figure 2A) and protein levels (Figure 2B). As in untransfected cells, a progressive decline in ferritin and a corresponding increase in NCOA4 in response to DFO treatment occurred in control cells (cells receiving scrambled siRNA) (Figure 2C). NCOA4 deficiency *per se* resulted in a ∼50% drop in ferritin abundance (Figure 2B). However, the response of ferritin to iron restriction was absent in NCOA4-depleted cells (Figure 2C). Accordingly, ferritin levels in NCOA4-deficient cells ended up being higher than those in control cells when both groups of cells were treated with DFO for 24 h. While DFO treatment or NCOA4 deficiency alone did not influence cell viability, the combination of the two led to morphological changes (Figure 2D) and compromised viability of HT22 cells (Figure 2E). Overall, these data indicate that NCOA4 plays a larger role in ferritin turnover in neurons under iron depletion, and ferritinophagy constitutes a primary route of iron supply when neuronal iron import is restricted.

### 3.3. Quantitative proteomic analysis reveals the role of NCOA4 in cellular iron homeostasis and mitochondrial function, with potential implications for neurodegenerative disease pathology

**FIGURE 3:**
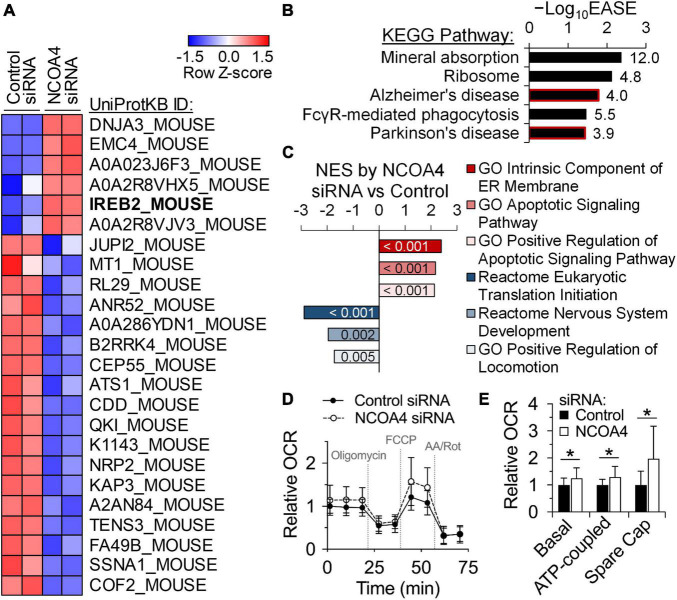
Quantitative proteomics identify roles of NCOA4 in apoptosis, translation, and neuronal functioning and development. Protein expression profiles of control and NCOA4-depleted HT22 cells were compared using the tandem mass tag quantitative proteomics approach. **(A)** Expression of proteins with FC values above 2.0 or below -2.0, and *P* < 0.05. Heatmap was generated with Z-scores of Log_2_-transformed normalized expression values. **(B)** KEGG Pathways enriched by DE proteins of NCOA4-depleted cells with |FC| > 1.5 and *P* < 0.05, which were identified using the DAVID functional annotation analysis. EASE Score is a modified Fisher’s exact *P*-value from the DAVID tool. Numbers next to each bar indicate fold enrichment for DE proteins. **(C)** Normalized enrichment scores (NES) of gene sets significantly enriched by proteins DE by NCOA4 depletion (*P* < 0.05) from GSEA. Numbers in each bar indicate *P*-value of enrichment by the DE proteins. **(D)** Effects of NCOA4 depletion on real-time OCRs after treatments of oligomycin, FCCP, and antimycin A/rotenone. Individual well OCR values were normalized to cell density. Data from *n* = 3 independent cultures and are presented as mean ± SD. **(E)** Basal respiration, ATP-coupled respiration, and spare respiratory capacity (Spare Cap) calculated from OCR measurements **(D)**. *P < 0.05 compared with control levels. FC, fold-change; KEGG, Kyoto Encyclopedia of Genes and Genomes; DE, differentially expressed; DAVID, Database for Annotation, Visualization, and Integrated Discovery; GSEA, Gene Set Enrichment Analysis; OCR, oxygen consumption rate.

Bioinformatic analysis of omics data permits prediction of the pathophysiological and organismal implications of *in vitro* treatments. To evaluate the roles of NCOA4 in the HT22 neuronal cell line, we performed proteomics analysis to determine differential expression patterns induced by the loss of NCOA4. Employment of quantitative proteomics as our initial omics analysis concerned the post-transcriptional regulatory mechanisms for genes involved in iron homeostasis and storage (4, 7, 8). Of relevance are NCOA4 and ferritin, which primarily respond to iron status at the protein but not the transcript level (7, 8). Remarkably, the iron regulatory protein IRP2 (IREB2_MOUSE) was identified as one of the proteins upregulated the most in response to NCOA4 depletion (Figure 3A). The turnover of IRP2 is facilitated by cytosolic iron in a proteasome-dependent manner (4). Thus, elevated IRP2 abundance is indicative of functional iron deficiency. Additionally, the decline in ferritin expression induced by NCOA4 deficiency, which was initially identified by western blot analysis (Figure 2B), was confirmed by lower counts of FTH and FTL peptides in the proteomics dataset (Supplementary Dataset 1).

To ascertain the pathophysiological implications of the differential expressions by NCOA4 deficiency, proteins with significant increases of more than 1.5-fold were subjected to bioinformatic pathway analyses. Over-representation analysis using the DAVID tools identified significant enrichment of gene sets associated with mineral absorption, ribosomes, and the neurodegenerative disorders, Alzheimer’s disease, and Parkinson’s disease (Figure 3B and Supplementary Table 2). Functional class sorting by the Gene Set Enrichment Analysis (GSEA) of upregulated proteins revealed “endoplasmic reticulum membrane” and “apoptotic signaling pathway” as the enriched gene sets in NCOA4-deficient cells (Figure 3C and Supplementary Figure 1). The reactome of nervous system development and positive regulation of locomotion were among the gene sets enriched in the analysis of proteins significantly downregulated by NCOA4 depletion (Figure 3C and Supplementary Figure 1).

One of the main roles of iron in cell biology is regulation of mitochondrial oxidative phosphorylation by providing prosthetic groups for TCA cycle and electron transport chain enzyme protein subunits. The list of proteins significantly upregulated by the loss of NCOA4 included proteins integral to mitochondrial function and respiration (Supplementary Dataset 1). Of relevance were the proteins DnaJ homolog subfamily A member 3 (DNAJA3), cytochrome c oxidase subunit 2 (COX2), dihydrolipoyllysine-residue acetyltransferase component of pyruvate dehydrogenase complex (DLAT), and the mitochondrial fission 1 protein (FIS1). Thus, we determined if the expression of NCOA4 affects real-time cellular oxygen consumption rate (OCR) in HT22 cells (Figure 3D). DFO treatment decreased all measures of mitochondrial-specific respiration (Supplementary Figure 2). Combining DFO treatment with *Ncoa4* knockdown did not alter the magnitude or direction of the DFO effects (Supplementary Figure 2). However, *Ncoa4* knockdown alone increased all aspects of mitochondrial respiration including basal, ATP-coupled and maximal respiration (Figure 3E), demonstrating the functional relevance of the increased mitochondrial protein abundance quantified by proteomics. The spare respiratory capacity of HT22 cells was also increased by NCOA4 knockdown, indicating an elevated ability to respond to metabolic stress (Figure 3E). Non-mitochondrial respiration, coupling efficiency, and proton leak levels were not altered.

### 3.4. Loss of NCOA4 impairs the response of IRP2 to iron restriction in HT22 cells

**FIGURE 4:**
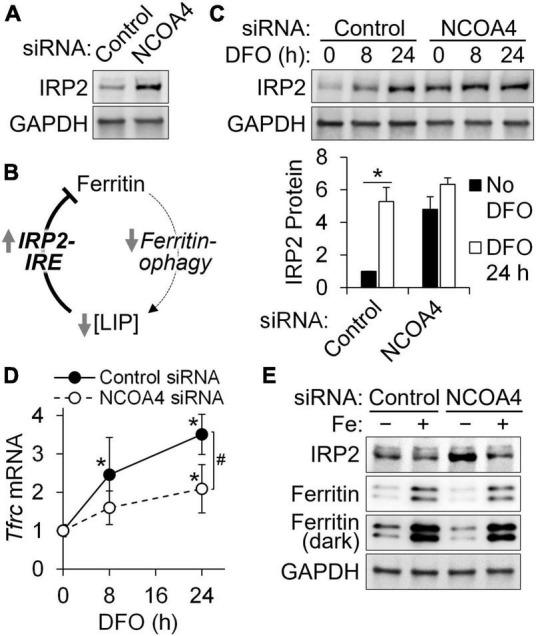
NCOA4 depletion impairs the adaptive responses of iron genes to cellular iron restriction in HT22 cells. HT22 cells were treated with *Ncoa4* siRNA to knockdown NCOA4 expression. **(A)** Accumulation of IRP2 protein by NCOA4 depletion. **(B)** Schematic model of IRP2-mediated ferritin repression by NCOA4 depletion. **(C)** Loss in IRP2 responses to iron restriction in NCOA4-depleted cells. DFO was added at 100 μM. IRP2 quantitation was normalized to GAPDH. **(D)** NCOA4 depletion represses the responses of *Tfrc* mRNA to iron restriction (DFO, 100 μM). Transcript abundance is relative to control siRNA-treated cells without DFO, and was normalized to that of *Tbp*. **(E)** IRP2 and ferritin remain responsive to supplemental iron in NCOA4-depleted HT22 cells. Iron was added as ferric ammonium citrate at 200 μM for 24 h. Data in panels **(C,D)** are presented as mean ± SD, and are from *n* = 3–5 independent experiments. *P < 0.05 by DFO; ^#^*P* < 0.05 by NCOA4 depletion. DFO, desferrioxamine; LIP, labile iron pool.

The accumulation of IRP2 by NCOA4 depletion, which had been initially identified by quantitative proteomic analyses (Figure 3A), was confirmed by western analyses (Figure 4A). An E3 ubiquitin ligase, FBXL5, promotes IRP2 turnover when the cytosolic LIP expands (28). Conversely, a reduction in LIP results in the upregulation of IRP2, which represses the translation of ferritin transcripts by binding to their IRE. Thus, the net reduction in ferritin (despite impaired ferritinophagy) by NCOA4 depletion could be attributed to the concomitant IRP2 activity (Figure 4B). Next, we determined how DFO-mediated restriction of iron import influences IRP2 in NCOA4-deficient cells. DFO treatment induced a progressive accumulation of IRP2 in control cells (treated with scrambled siRNA), reaching a 5-fold increase after 24 h of treatment (Figure 4C). However, IRP2 abundance did not change in response to DFO treatment in NCOA4-deficient cells. The mRNA of *Tfrc* contains five IREs at its 3′-UTR, and thus is stabilized by increased IRP activity. In agreement with the lack of IRP2 regulation by DFO in NCOA4-deficient cells, *Tfrc* transcript abundance no longer responded to iron restriction when cells were deficient of NCOA4 (Figure 4D). Notably, *Tfrc* transcript abundance was not affected by NCOA4 depletion when the cells did not receive DFO (Figure 4D), despite their higher IRP2 levels (Figure 4A). Unlike iron restriction, iron supplementation produced a change in IRP2 levels in both control and NCOA4-depleted HT22 cells (Figure 4E).

### 3.5. Functional enrichment analyses of the transcriptome of NCOA4- and iron-deficient HT22 cells reveal neurological implications of ferritinophagy during iron deficiency

For a more comprehensive assessment of the pathophysiological implications of the interactions between ferritinophagy and neuronal iron status, the transcriptome of HT22 cells deficient in NCOA4, iron import, or both were profiled *via* RNA-seq and analyzed bioinformatically. Initially, we compared the transcriptome and proteome profiles of control and NCOA4-deficient cells to identify 64 genes responsive to NCOA4 depletion at both transcript and protein levels (Supplementary Figure 3). Functional network analyses of transcriptome data revealed neurological disease and nervous system development and function as part of the functional networks associated with NCOA4 deficiency (Supplementary Figure 4).

**FIGURE 5:**
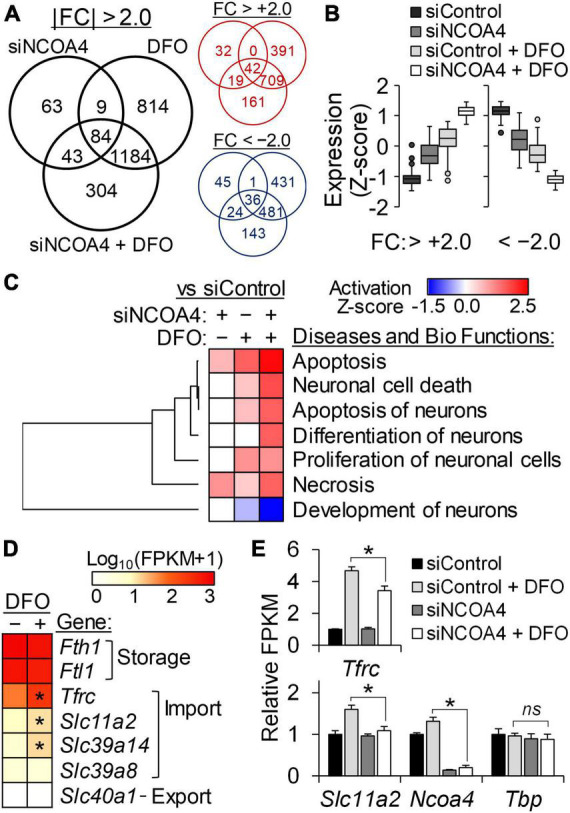
Transcriptome analysis reveal a protective role of NCOA4 against apoptosis induced by neuronal iron deficiency. Transcripts responsive to NCOA4 depletion by siRNA (siNCOA4), iron deficiency (DFO), and a combination of the two (siNCOA4 and DFO) were identified by RNA-seq (*n* = 3 biological replicates). DFO was treated at 100 μM for 24 h. **(A)** Numbers of differential expressions shared by or exclusive to each treatment are shown in the black Venn diagram. Transcripts with | FC| > 2.0 and FDR-adjusted *P* < 0.05 vs control siRNA (siControl) were considered DE. Venn diagrams in red and blue indicate the numbers of upregulated and downregulated genes, respectively, by each treatment. **(B)** Expression pattern of DE genes exclusively identified in cells treated with siNCOA4 plus DFO. For each gene, expression values of all treatment groups were standardized by Z-score transformation to yield a mean of zero and SD of one. **(C)** Comparisons among the IPA activation Z-scores of neuron-related Diseases and Bio Functions by genes responsive to siNCOA4, DFO, and siNCOA4 plus DFO. IPA core analyses was performed for DE by each treatment (vs siControl), and further analyzed by comparison analysis. Neuron-related Diseases and Bio Functions with significant activation Z-scores by siNCOA4 plus DFO vs siControl are shown (*P* < 0.05). **(D)** Relative transcript abundance of genes for cellular iron homeostasis determined by RNA-seq FPKM. Gene expression data was ranked by mean FPKM values of siControl cells, and presented as Log_10_(FPKM + 1). *P < 0.05 by DFO. **(E)** Less *Tfrc* and *Slc11a2* transcript responses to iron restriction in NCOA4-depleted cells. Data are shown as mean ± SD of FPKM from *n* = 3 biological replicates. *P < 0.05 by NCOA4 depletion; ns, not significantly different; siNCOA4, *Ncoa4* siRNA; siControl, control siRNA; DFO, desferrioxamine; DE, differentially expressed; FC, fold-change; FDR, false discovery rate; vs, versus.

Among the entire RNA-seq dataset, the set of genes differentially regulated in HT22 cells deficient in both ferritinophagy and iron import, a condition which led to cytotoxicity, was of particular interest. A sum of 304 transcripts with fold-changes (FC) above 2 and adjusted FDR *q*-values below 0.05 were identified exclusively in cells treated with *Ncoa4* siRNA and DFO together (Figure 5A). The expression trend of these genes suggests that ferritinophagy can support neurons in attenuating the magnitude of gene responses produced by restricted iron import, and vice versa (Figure 5B). Neuronal cell death, apoptosis, and differentiation of neurons were among the diseases and biofunctions predicted as activated by the simultaneous losses in ferritinophagy and iron import in HT22 cells (Figure 5C). Neuronal development pathways of neurons were affected in the opposite manner, indicating NCOA4-mediated ferritinophagy is necessary for gene programs important for normal iron-dependent neuron development.

The normalized gene expression values of iron homeostasis genes determined by RNA-seq indicated higher expression values of genes involved in cytosolic iron storage, *Fth1* and *Ftl1*, than those involved in transmembrane transport of the metal in HT22 cells. Among these, the transcript levels of iron importers *Tfrc*, *Slc11a2*, and *Slc29a14* were found to be iron responsive (Figure 5D). Notably, the iron exporter gene *Slc40a1* did not produce detectable amounts of transcripts in HT22 cells regardless of the cellular iron status. The gene *Slc11a2* encodes the divalent metal transporter 1 (DMT1) protein which transports iron across cellular membranes into the cytosol. Like *Tfrc*, *Slc11a2* transcripts carry 3′-UTR IREs and thus are stabilized by iron restriction *via* IRP binding. Supporting our previous observation on the lack of IRP2 regulation by DFO in NCOA4-deficient cells (Figure 4C), the transcripts of both *Tfrc* and *Slc11a2* were identified less responsive to iron restriction by the RNA-seq data of NCOA4-depleted HT22 cells (Figure 5E).

## 4. Discussion

Iron is an essential nutrient crucial for neurodevelopment and lifespan brain health (29). Conversely, excessive accumulation of iron and ferritin in the brain has been associated with the pathogenesis of neurodegeneration and neuronal loss (30, 31). Thus, neurons need a regulatory system which allows them to efficiently balance their iron availability with their biological demand for the metal nutrient. Using HT22 cells as an *in vitro* cell line model of mouse hippocampal neurons, we identified ferritinophagy as a potential mechanism by which neurons maintain a balance between their supply and demand for iron, especially when iron becomes scarce. The importance of ferritin at the cellular level is twofold. First, it sequesters excess iron, which can be potentially toxic, and stores it for future use. The second function of ferritin as an iron resource is strongly supported herein, and the elevated IRP2 and increased sensitivity to cellular iron restriction by NCOA4 depletion are of particular relevance.

The transcriptomic and proteomic profiles of NCOA4 deficiency in HT22 cells revealed enrichment of gene responses functionally associated with neurobiology and neurological disorders, and their interaction with cellular iron status. These included neuronal cell death, neurodevelopment, neuron proliferation, and neurodegeneration. The bioinformatic association between impaired ferritinophagy and molecular pathways of neurodegenerative conditions agree with previous preclinical observations linking functional iron deficiency of neurons to neurodegeneration. Particularly, *Irp2*-null mice feature progressive neurodegeneration attributed to neuronal iron restriction (32). The genetic loss of IRP2 misinforms neurons as if they are in iron excess. This leads to changes in iron homeostatic gene expressions driving a decline in the cellular import and more removal *via* export and storage (4), and thus functional iron deficiency. Additionally, conditional deletion of *Tfrc* in dopaminergic neurons of mice resulted in neurological and behavioral phenotypes recapitulating symptoms of Parkinsonism in humans (33). These and our *in vitro* data collectively inform the need for future studies using primary neurons and an *in vivo* model of neuronal *Ncoa4* deficiency, testing whether functional iron deficiency by impaired ferritinophagy similarly influences the central nervous system and produces phenotypes of neurodegeneration.

Early iron deficiency has been shown to impair learning and memory, which has been attributed to disrupted mitochondrial function and neuronal loss (21). Our quantitative omics analyses revealed differential expressions associated with mitochondrial function and apoptosis by NCOA4 deficiency. Of particular relevance were DNAJA3, COX2, and apoptotic protease-activating factor 1 (APAF). DNAJA3 modulates apoptotic signal transduction within the mitochondrial matrix and has been characterized as a metalloprotein (34). Known for its role in mitochondrial energy transduction, cytochrome c oxidase has been suggested to play a role in stress-induced apoptosis and degenerative diseases (35). APAF1 is involved in apoptosis due to its binding and subsequent activation of procaspase-9, and studies suggest that cytochrome c and APAF1 work together to activate the apoptotic pathway (36). NCOA4 is a multifunctional protein; thus, the differential expression patterns induced by its loss in HT22 neurons cannot not be attributed solely to its role in ferritinophagy. However, morphological changes, compromised cell viability, and transcriptome profiles of neurodegeneration by NCOA4 depletion were augmented or introduced by coexisting iron deficiency. These indicate a clear nutrient-gene interaction pattern where cellular dependence on NCOA4 becomes more apparent under conditions of insufficient nutrient supply. On the other hand, NCOA4 depletion and iron restriction by DFO had opposite effects on mitochondrial function measured by OCR. Future mechanistic studies with primary neurons need to be performed to specifically determine whether neuronal iron mediates regulation of mitochondrial function by NCOA4.

Ferroportin can contribute to the removal of iron from the cytosol as an iron exporter on the plasma membrane. Interestingly, the transcripts for the iron exporter ferroportin (*Slc40a1*) were undetectable by our RNA-seq experiments, which was in contrast to the relatively higher counts for the ferritin (*Fth1* and *Ftl1*) transcripts. Ferroportin is the sole non-heme iron exporter on the plasma membrane. Thus, the lack of ferroportin expression in HT22 cells suggests that sequestration of iron by ferritin serves as the primary mode of protection against excess cytosolic iron in neurons. Alternatively, excess iron might be removed from HT22 cells *via* a ferroportin-independent route, such as exosomal ferritin as recently described (37). These are in agreement with previous *in vivo* findings where *Slc40a1* was identified dispensable for the survival and function of dopaminergic neurons in mice (33).

Collectively, our studies using HT22 cells identify how NCOA4 is a critical nexus regulating the tight control of intracellular iron to maintain cell viability. NCOA4-mediated ferritinophagy was identified to be crucial for adaptation to fluctuations in iron levels, particularly when cellular iron supply is restricted. Loss of NCOA4 led to molecular marker changes implying functional iron deficiency and differential gene expressions associated with development of neurons, mitochondrial function, apoptosis, and neurodegenerative disorders. Our *in vitro* data suggest that the cellular iron pool and NCOA4-mediated ferritinophagy could serve as potential molecular targets for prevention of neurodegenerative disorders and facilitate the development of therapeutic strategies for their treatment. Future studies on primary neurons and the *in vivo* brain are needed to understand the requirement of ferritinophagy for brain development and function throughout the lifespan, and to decipher the mechanisms underlying the genetic and nutritional regulation of NCOA4 expression and activity in the central nervous system.

## Data availability statement

The original contributions presented in this study are included in this article/Supplementary material, further inquiries can be directed to the corresponding author. The RNA-seq data presented in this study are deposited in the NCBI Gene Expression Omnibus (GEO) repository, accession number GSE211931 (https://www.ncbi.nlm.nih.gov/geo/query/acc.cgi?acc=GSE211931).

## Author contributions

M-SR: conceptualization and supervision. EB, TB, and M-SR: methodology and investigation. EB and M-SR: formal analysis, data curation, original draft preparation, and visualization. CG: validation. MG and M-SR: resources and project administration. CG, TB, MG, and M-SR: review and editing. All authors have read and agreed to the published version of the manuscript.
