# Supplementary material for: Quantitative omics analyses of NCOA4 deficiency reveal an integral role of ferritinophagy in iron homeostasis of hippocampal neuronal HT22 cells
Source: Front Nutr. 2023 Jan 19;10:1054852. doi: 10.3389/fnut.2023.1054852 (PMC9892431; doi:10.3389/fnut.2023.1054852)
Supplement: Supplementary file 1 [file Data_Sheet_1.DOCX]

Supplementary Material

**Supplementary Table 1.** Primer pairs for SYBR Green-based qPCR analyses. Custom primers specific to each gene transcript were designed using Primer-BLAST (<https://www.ncbi.nlm.nih.gov/tools/primer-blast/>).

| Gene | Primer Sequence | | T_m_ (°C) | GC% | Amplicon |
| --- | --- | --- | --- | --- | --- |
| *Tfrc* | Forward: | 5’-TCACTTCCTGTCGCCCTATGT-3’ | 60.9 | 52.4 | 78 bp |
|  | Reverse: | 5’-AGAGTGTGAGAGCCAGAGCC-3’ | 61.3 | 60.0 |  |
| *Ncoa4* | Forward: | 5’-AGCTAAGGCACCCAAGGCTA-3’ | 60.9 | 55.0 | 115 bp |
|  | Reverse: | 5’-CTTAGGGCCTCCTTTGCACG-3’ | 61.0 | 60.0 |  |
| *Tbp* | Forward: | 5'-AGTTGTGCAGAAGTTGGGCT-3' | 60.1 | 50.0 | 128 bp |
|  | Reverse: | 5'-TACTGAAGGCTGGTGGGTCA-3' | 61.0 | 52.4 |  |

**Supplementary Table 2.** Functional annotation enrichments identified via DAVID analyses with proteins differentially expressed by NCOA4 depletion. Functional categories with an EASE score lower than 0.05, and a count of associated proteins above three are shown. The EASE score is a modified Fisher’s exact *P*-value by the DAVID tool.

| Category | Term | Count | Proteins | EASE | Fold Enrichment |
| --- | --- | --- | --- | --- | --- |
| Gene Ontology: Biological Process | Regulation of translation | 5 | ZFP385A, RBM3, IREB2, TNRC6A, QK | 8.15E-03 | 6.28 |
|  | Cell migration | 6 | TNS3, CTGF, PDGFRA, MMP14, CD151, SIRPA | 8.35E-03 | 4.77 |
|  | Ribosomal large subunit biogenesis | 3 | RPL14, NPM1, NOC2L | 1.06E-02 | 18.99 |
|  | Iron ion transport | 3 | FTL1, IREB2, FTH1 | 1.06E-02 | 18.99 |
|  | Regulation of exocytosis | 3 | SEPT5, RALA, PCLO | 1.15E-02 | 18.23 |
|  | Apoptotic process | 10 | CSNK2A2, FIS1, ZFP385A, EMC4, DAD1, APAF1, DNAJA3, RTN3, WDR92, NOC2L | 1.24E-02 | 2.67 |
|  | Negative regulation of cardiac muscle cell apoptotic process | 3 | NPM1, AMBRA1, QK | 1.24E-02 | 17.53 |
|  | Branching morphogenesis of an epithelial tube | 3 | SEMA5A, MMP14, PXN | 2.30E-02 | 12.66 |
|  | Hematopoietic progenitor cell differentiation | 4 | EML1, PDGFRA, PRRC2C, SIRPA | 2.63E-02 | 6.20 |
|  | Cellular iron ion homeostasis | 3 | FTL1, IREB2, FTH1 | 3.48E-02 | 10.13 |
|  | Positive regulation of cysteine-type endopeptidase activity involved in apoptotic process | 3 | FIS1, CTGF, ARRB1 | 3.92E-02 | 9.50 |
|  | Learning or memory | 3 | NCAM1, ZFP385A, AMFR | 4.38E-02 | 8.94 |
|  | Lung development | 4 | CTGF, PDGFRA, PTN, MMP14 | 4.76E-02 | 4.90 |
| KEGG Pathway | Mineral absorption | 4 | FTL1, MT2, MT1, FTH1 | 4.24E-03 | 11.95 |
|  | Ribosome | 6 | RPS25, RPL14, RPL8, RPS21, UBA52, RPL29 | 7.41E-03 | 4.82 |
|  | Alzheimer's disease | 6 | ATP5E, NDUFA2, COX2, PPP3CA, APAF1, CAPN1 | 1.66E-02 | 3.95 |
|  | FcγR-mediated phagocytosis | 4 | ARPC3, CFL2, MARCKS, VASP | 3.38E-02 | 5.55 |
|  | Parkinson's disease | 5 | SEPT5, ATP5E, NDUFA2, COX2, APAF1 | 3.68E-02 | 3.91 |

**
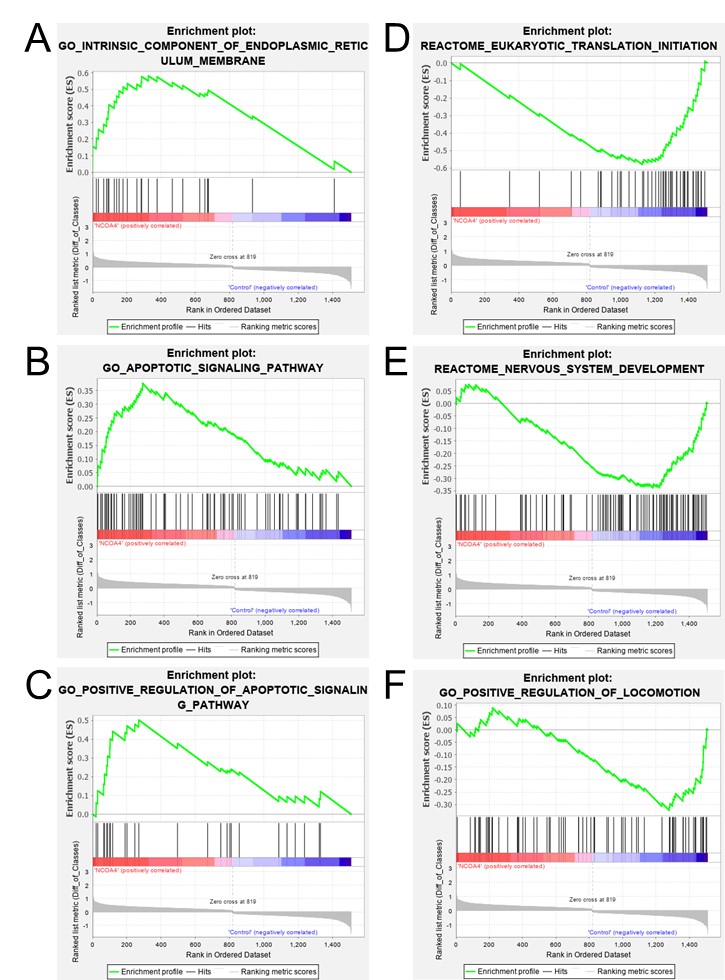
**

**Supplementary Figure 1.** GSEA enrichment plots of functional categories enriched by proteins responsive to NCOA4 depletion in HT22 cells. GSEA was performed with the quantitative proteomics data of proteins differentially expressed by NCOA4 depletion (*P* < 0.05). (**A-C**) Enrichment plots of gene sets enriched by proteins upregulated by NCOA4 siRNA vs control siRNA: GO Intrinsic Component of ER Membrane (**A**), GO Apoptotic Signaling Pathway (**B**), GO Positive Regulation of Apoptotic Signaling Pathway (**C**). (**D-F**) Enrichment plots of gene sets enriched by proteins downregulated by NCOA4 siRNA versus control siRNA: Reactome Eukaryotic Translation Initiation (**D**), Reactome Nervous System Development (**E**), GO Positive Regulation of Locomotion (**F**).


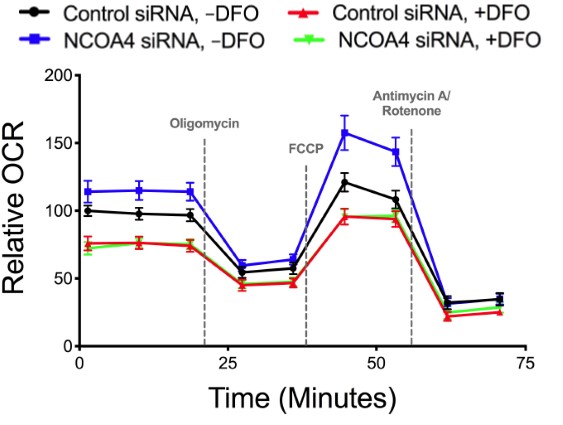


**Supplementary Figure 2.** Effects of cellular iron restriction on the relative oxygen consumption rates (OCR) of control and NCOA4-depleted HT22 cells. HT22 cells were transfected with control or NCOA4 siRNA, and treated with or without DFO (100 µM) for 24 h. Real-time OCR was measured following treatments with oligomycin, FCCP, and antimycin A/rotenone.

**Supplementary Figure 3.** Differentially expressed proteins with significant changes in transcript abundance. Proteins identified by quantitative proteomics were filtered by |Fold-change (FC)| above 1.5, and *P*-value below 0.05 for significance. Among the genes encoding proteins with significant differential expression by NCOA4 depletion, 64 featured changes in transcript abundance by RNA-seq (FDR-adjusted *P* < 0.05). (**A**) Genes featuring differential expressions by NCOA4 depletion at both protein and mRNA levels. (**B**) Association between the protein and mRNA responses of genes in **A**. Modes of mRNA responses (up versus down) to NCOA4 depletion (siNCOA4) corresponded to those of the protein.

**Supplementary Figure 4.** Top functional networks of genes, which are differentially expressed in HT22 cells with losses of both NCOA4 and iron. Transcriptome profiles of HT22 cells transfected with NCOA4 siRNA and treated with DFO (100 µM) were compared with Control siRNA-treated cells. Functional implications of the gene responses were predicted using the Ingenuity Pathway Analysis (IPA). (**A**) Top 5 functional networks affected by the combined losses of NCOA4 and iron in HT22 cells. (**B**) The top functional network of differential expressions associated with cancer, neurological disease, and organismal injury and abnormalities. Histone H3 and HIF1 were identified as potential regulatory nodes mediating the responses of genes to the combined losses of NCOA4 and iron.

**Supplementary Dataset 1. Proteins identified via tandem mass tag (TMT) quantitative proteomics.**

**Supplementary Dataset 2. Differential expressions by NCOA4 siRNA, DFO, and a combination of the two determined by RNA-seq.**
